# Advance Microbiota Transplantation: A Novel Addition–Subtraction Paradigm for Optimising Faecal Microbiota Transplantation

**DOI:** 10.1111/1751-7915.70323

**Published:** 2026-03-10

**Authors:** Haojia Lin, Zelin Feng, Qiuyue Tu, Huizhen Li, Yanru Zhang, Xinyue Wei, Qinghua Yi, Hetong Zhang, Yu Wang, Xiaoqin Li, Yueting Li, Jun Huang, Zehan Chen, Hongtian Shentu, Anjiang Wang, Ye Chen, Xiaolong He, Xiaocang Cao

**Affiliations:** ^1^ Department of Gastroenterology and Hepatology Tianjin Medical University General Hospital Tianjin China; ^2^ Tianjin Medical University Tianjin China; ^3^ Department of Gastroenterology, Integrative Microecology Clinical Center, Shenzhen Key Laboratory of Gastrointestinal Microbiota and Disease, Shenzhen Clinical Research Center for Digestive Disease, Shenzhen Technology Research Center of Gut Microbiota Transplantation, Shenzhen Hospital Southern Medical University Shenzhen China; ^4^ Department of Gastroenterology, State Key Laboratory of Organ Failure Research, Guangdong Provincial Key Laboratory of Gastroenterology Nanfang Hospital, Southern Medical University Guangzhou China; ^5^ Microbiome Medicine Center, Department of Laboratory Medicine, Zhujiang Hospital Southern Medical University Guangzhou Guangdong China; ^6^ Guangdong Provincial Clinical Research Center for Laboratory Medicine Guangzhou Guangdong China

**Keywords:** addition–subtraction strategy, advance microbiota transplantation, faecal microbiota transplantation, preferred reporting items for microbiotherapy (PRIM)

## Abstract

Faecal microbiota transplantation (FMT) is highly effective for recurrent *Clostridioides difficile* infection but yields inconsistent benefits in chronic indications. As a crude whole‐microbiota transplant, FMT contains numerous undefined active components, complicating efforts to ensure treatment predictability and stability. Therefore, we propose Advance Microbiota Transplantation (AMT), a comprehensive, phase‐based hypothesis that employs an addition–subtraction strategy throughout the pre‐, peri‐ and post‐transplant stages. AMT comprises donor and recipient pre‐treatment, procedural optimisation and post‐transplant adjuvant interventions to mitigate donor variability, ecological resistance, procedural heterogeneity and unstable engraftment. Through a systematic synthesis of current evidence‐based FMT research, we explored how the addition–subtraction strategy can be operationalised to shape the AMT concept and define testable, phase‐specific levers, thereby providing a foundation for future clinical translation. In parallel, we appraised the reporting quality using the Preferred Reporting Items for Microbiotherapy (PRIM) and identified six persistently under‐reported items that limit the interpretability, comparability, and reproducibility of FMT research. This review aims to facilitate the integration of AMT into clinical practice.

## Graphical abstract

This review proposes Advance Microbiota Transplantation (AMT), a pre‐peri‐post transplant ‘addition–subtraction’ framework that integrates donor–recipient optimisation, product engineering, and post‐transplant recipient adjuvant management to enhance conventional FMT efficacy and mitigate its limitations.

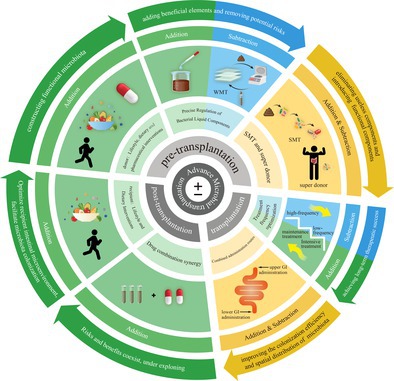

## 1 Introduction

Faecal microbiota transplantation (FMT) has transitioned from a salvage therapy for recurrent *Clostridioides difficile* infection (rCDI; van Nood et al. 2013; Cammarota et al. 2017) to an investigational therapy for chronic, immune‐ and metabolism‐related diseases (Wu et al. 2022). Advances in product handling and delivery, including washed microbiota transplantation (WMT) and transendoscopic enteral tubing, have reduced risks, improved administration precision, and accelerated clinical adoption (Gulati et al. 2020; Lu et al. 2022).

However, the advancement of FMT has encountered several obstacles. Donor availability and interindividual variability hinder reliable access to ‘high‐quality’ microbiota; the recipient gut often presents ecological resistance—competition for nutrients and space—that impedes engraftment; technical non‐uniformity persists across the field: preparation standards (oxygen exposure, storage, cryoprotectants), delivery routes (e.g., colonoscopy, enema, oral capsules), and treatment protocols (frequency, dose) vary without a stable evidence base; colonisation stability is difficult to maintain as host communities rebound and regulatory classification is fragmented across jurisdictions, complicating product development and trial comparability. Together, these limitations help explain the variable clinical responses and slow progress toward standardisation.

To address these gaps, we propose Advance Microbiota Transplantation (AMT), an ‘addition–subtraction’ paradigm that treats FMT as a multiphase therapeutic process. AMT operationalises targeted levers before, during, and after transplantation: pre‐transplant donor enhancement and recipient ‘de‐resistance’ (addition/subtraction), peri‐transplant optimisation of frequency, route, and dose, and post‐transplant microenvironment management via diet, drugs, and lifestyle to consolidate the engraftment. This review does not assert clinical validation of AMT but systematically synthesises current evidence‐based FMT research, exploring how the addition–subtraction strategy can be operationalised to shape the AMT concept.

In parallel, we evaluated recent clinical studies using the PRIM reporting framework. This audit highlights persistent deficiencies in donor screening, production methods, product classification, excipients, patient preparation, and concomitant treatments, which are critical for interpretability and replication. Integrating AMT's process‐based design with PRIM‐guided reporting provides a practical pathway toward standardisation: it clarifies which levers matter, when to deploy them, and how to report them so that findings are comparable across studies.

By systematically unpacking AMT's multi‐phase optimisation logic of AMT and addressing reporting gaps via PRIM, this review aims to lay a foundation for translating AMT from a conceptual framework to clinical practice and ultimately promote the shift of microbiota transplantation from empirical to precision therapy.

## 2 Bibliometric Evaluation of FMT Studies and Quality Assessment of Reports

This study retrieved English‐language clinical studies on FMT from PubMed, Embase, and the Cochrane Library covering the period from January 2016 to July 2025 (search strategy is presented in File S1). Following the PRIM guidelines (Zhang, Kamm, et al. 2025; Zhang, Liu, et al. 2025), two reviewers independently screened the titles and abstracts after deduplication for quality assessment. The inclusion criteria were as follows: (1) human studies; (2) patients treated with FMT (including novel microbiota products such as SER‐109); and (3) publications in English. The exclusion criteria were as follows: (1) non‐original research (e.g., reviews, conference abstracts); (2) full‐text unavailable; (3) reports analysing biological samples related to FMT (including microbiome sequencing, metabolomics, and genetic assessments) and (4) duplicated articles. Full‐text reviews were conducted for abstracts that achieved consensus approval, and any discrepancies were resolved through discussion. Independent dual full‐text screening and data extraction captured study characteristics, including design, publication year, country, sample size, and PRIM checklist items. Statistical analyses were performed using IBM SPSS 23.0 and GraphPad Prism 8.0, with non‐normally distributed continuous variables analysed using the Mann–Whitney *U* test or Kruskal‐Wallis test (with Dunn's post hoc analysis; *α* = 0.05).

**FIGURE 1:**
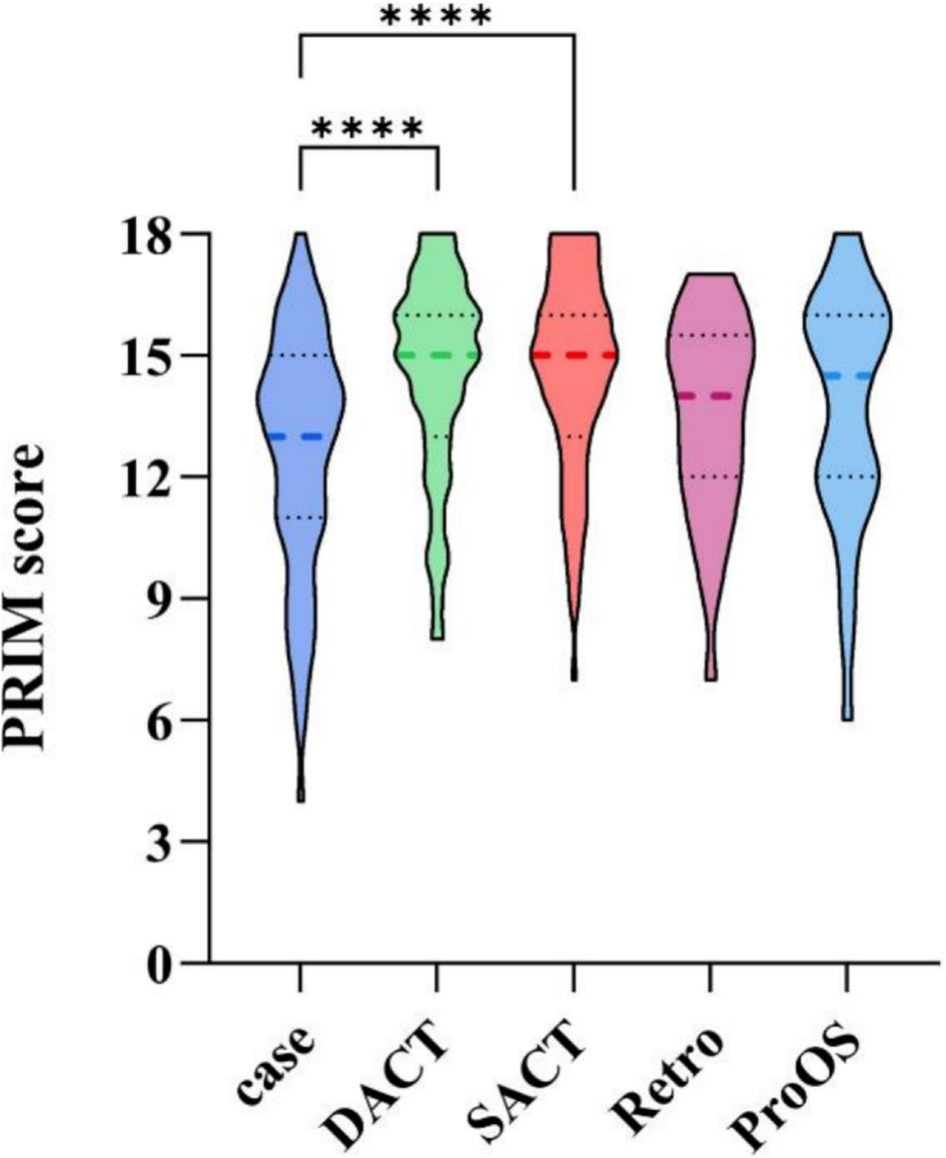
The PRIM scores comparison of different research types (****p < 0.0001). DACT, double‐arm clinical trial; Retro, retrospective study; ProOS, prospective observational study; SACT, single‐arm clinical trial.

**FIGURE 2:**
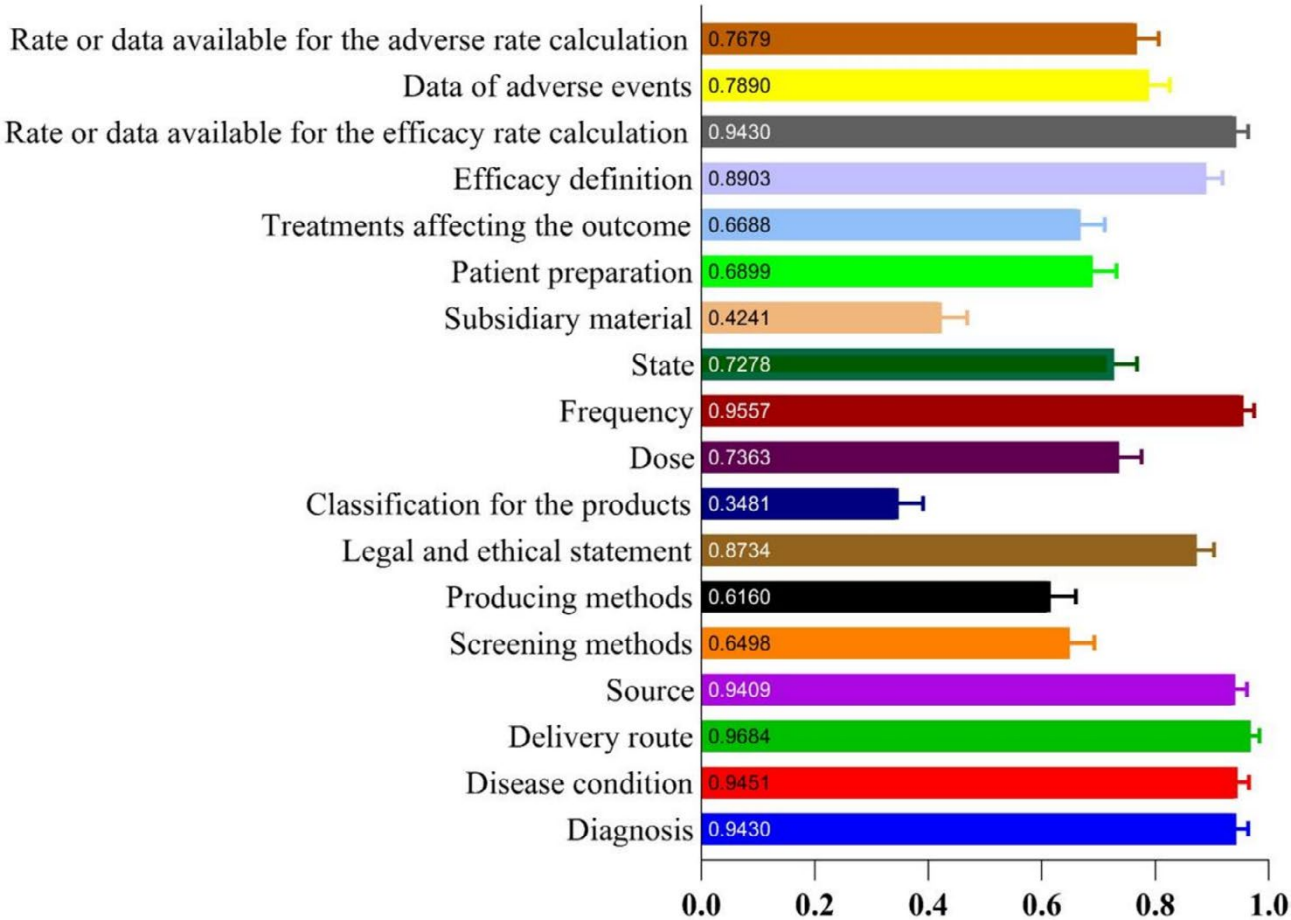
Average score of each item in PRIM.

The screening workflow is shown in Figure S1. FMT research output has increased over time, with a clear shift from case reports to clinical trials, reflecting a move from anecdotal to evidence‐based designs. Across the corpus, 63.5% of studies met high‐quality thresholds (≥ 14 points), whereas 13.5% were low‐quality (≤ 10 points, Figure S2), and trials scored significantly higher than case reports (adjusted *p* < 0.0001, Figure 1). Item‐level appraisal revealed persistent weaknesses in six domains: donor screening methods, production methods, product classification, subsidiary/excipient materials, patient preparation, and concomitant treatments affecting outcomes (Figure 2; detailed statistics in Table S1).

Research volume and quality jointly indicate the field's maturation, as publication growth reflects a shift from anecdotal evidence to evidence‐based designs. Although contemporary clinical trials generally follow rigorous reporting standards, case reports still omit essential information. This gap likely stems from protocol preregistration and guideline adherence in trials versus the narrative emphasis and lack of uniform templates in case reports, which undermine interpretability, reproducibility, and clinical translation. Item‐level weaknesses clarify where standardisation is most necessary. Product classification suffers from ambiguous legal categories (‘drug’, ‘investigational new drug’, ‘medical technology’) and heterogeneous ethics requirements across jurisdictions, complicating compliance and cross‐regional comparisons and risking obscured ethical issues. Subsidiary materials are often under‐reported, indicating that studies overlook their potential impact on the microbial community and recipient health. Moreover, there are no standardised guidelines for common agents (saline, glycerin, cryoprotectants), leading to vague reporting that hinders safety assessments and product consistency. Donor screening may rely on opaque institutional criteria, potentially omitting critical safety indicators from consideration. Production methods (e.g., manual grinding, mechanical washing and freeze‐drying) are reported with unclear terminology, reducing reproducibility. Patient preparation (e.g., antibiotics, bowel cleansing) is frequently reduced to ‘standard’, ignoring baseline effects. Concomitant treatments (e.g., probiotics and diets) are insufficiently documented, obscuring interactions and outcome attribution.

Collectively, low scores across these items indicate that current FMT research lacks a standardised reporting framework across the study continuum and, as a result, often emphasises efficacy over methodological rigour. This undermines reproducibility, may obscure safety risks, and introduces bias. To address these issues, a shift in perspective is essential. FMT must be conceptualised as an integrated intervention system that incorporates microbial composition, procedural details, and environmental factors, as advanced by the ‘full‐process optimisation framework’ of AMT. To translate these heterogeneous findings into actionable insights for AMT, we mapped representative evidence‐based FMT trials onto the full AMT transplant cycle. We framed the interventions of these FMT trials as potential ‘addition‐subtraction operations’ and classified their implications as ‘suggestive’ or ‘negative’ evidence for refining AMT strategies (Table 1). This evidence mapping provides direct support for phase‐specific optimisation of AMT strategies, which we will detail in subsequent sections.

**TABLE 1:** Evidence mapping of addition–subtraction strategy in FMT: implications for advance microbiota transplantation (AMT).

AMT phase	Study	Indication	Specific additive/Subtractive operation	Strategy category	Major outcomes	AMT framework implications & evidence type
*Pre‐Donor*	Leibovitzh 2024, RCT	UC	Pre‐transplant anti‐inflammatory diet for donor; post‐transplant UCED for recipients	Addition	Faecal calprotectin↓; clinical remission rate↑	Supports donor diet conditioning for UC. [Suggestive]
	Smits 2018, pilot RCT	MD	vegan donor FMT	Addition	Microbiota altered; no TMAO/vascular inflammation markers improvement	Vegan donor alone insufficient. [Negative]
	Mizuno 2017, Open‐label pilot study	IBS	Bifidobacterium‐rich donor selection	Addition	60% clinical response; microbiota diversity↑	Suggests functional microbe‐enriched donors for IBS. [Suggestive]
	El‐Salhy 2020, RCT	IBS	Single super‐donor FMT	—	Symptom response at 3 mo; quality of life↑	Supports single super‐donor for IBS. [Suggestive]
Pre–Product	Feuerstadt 2022 & Straub 2023, phase 3 RCT	rCDI	Purified Firmicutes spore formulation	Addition	Recurrence↓; ARG abundance↓	Proves purified microbial formulations for rCDI. [Suggestive]
	Garza‐Gonzalez 2019, pilot RCT	rCDI	FMT + 3 Lactobacillus species	Addition	No benefit vs. standard FMT	Non‐specific probiotic enrichment unnecessary. [Negative]
	Chen 2020, open‐label trial	UC (moderate to severe)	WMT (multi‐step centrifugation + washing)	Subtraction	77.8% clinical response at wk. 2; 55.6% clinical remission at wk. 12	Supports WMT as safe subtractive lever. [Suggestive]
	Liang 2024, observational cohort	Hyperlipidemia			Serum lipid/ASCVD↓; no serious AEs	
	Xiao 2025, prospective observational study	Paediatric GI disease‐related malnutrition			48.3% response; GI symptoms relieved; mild self‐limiting AEs	
	Zhang 2023, prospective observational study	IBS			GI/extraintestinal symptoms improved; no severe AEs	
	Costello 2019, RCT	UC (moderate to severe)	Anaerobic processing of pooled stool	Subtraction	32% steroid‐free remission; diversity↑	Supports anaerobic processing to preserve anaerobes for UC. [Suggestive]
	Kao 2024, RCT	rCDI	Sterile faecal filtrate	Subtraction	Lower rCDI‐free rate vs. lyophilised FMT	Subtraction must preserve core microbes. [Negative]
	Rode 2021, RCT	rCDI	12‐strain bacterial mixture	Addi & Subtraction	52% clinical cure (inferior to FMT; superior to vancomycin)	Feasible but less effective than FMT for rCDI. [Suggestive]
	Kao 2021, phase 1 trial	rCDI	40‐strain microbial ecosystem (MET‐2)	Addi & Subtraction	79% recurrence‐free (Day 40); 95% (retreatment); 84% (Day 130)	Standardised multi‐strain additives safe/effective for rCDI. [Suggestive]
	Menon 2025, phase 2 RCT	rCDI	8‐strain consortium (VE303)	Addi & Subtraction	13.8% recurrence (vs 45.5% placebo)	Supports standardised multi‐strain additives for rCDI. [Suggestive]
	Halkjær 2018, RCT	IBS	4‐donor mixed stool	Addition	No symptom improvement vs. placebo	Mixed‐donor FMT offers no extra benefit for IBS. [Negative]
	Johnsen 2018, RCT	IBS	2‐donor mixed stool	Addition	65% symptom response (3mo, vs. 43% placebo); not maintained at 12mo	Mixed‐donor FMT has transient IBS efficacy, inconsistent long‐term outcomes. [Negative]
	Levast 2023, meta‐analysis	UC	Multi‐donor (MDN) vs. single‐donor (SDN)	Addition	MDN superior to SDN (RR = 2.81, *p* = 0.005)	Multi‐donor pooling is a superior additive lever for UC remission. [Suggestive]
Pre‐ Recipient	Gefen 2025, meta‐analysis	UC	Pre‐FMT biologic/steroid pretreatment	Addition	Clinical/endoscopic remission↑	Validates pre‐transplant drug pretreatment for UC. [Suggestive]
	van Lingen 2024, pilot RCT	UC	3‐week budesonide pretreatment	Addition	No engraftment/remission improvement vs. placebo	Budesonide pretreatment offers no value. [Negative]
	Chen 2020, retrospective study	Multiple indications (constipation, UC, IBS, rCDI, etc.)	Antibiotic/bowel cleansing pretreatment	Subtraction	Efficacy↑; adverse events↓ vs. no preparation	Supports pre‐transplant subtractive pretreatment. [Suggestive]
	Keshteli 2017, meta‐analysis of studies	UC	Antibiotic pretreatment	Subtraction	54% remission (vs 25.1% no antibiotics)	Supports antibiotic pretreatment for UC remission. [Suggestive]
Peri‐Dose & Route	Haifer 2022, RCT	UC	Induction + maintenance FMT	Addition	53% composite remission at wk. 8; 100% maintenance of remission at wk. 56	Supports sequential FMT for UC. [Suggestive]
	Paramsothy 2017, RCT	UC	High‐frequency + multi‐donor FMT	Addition	27% composite remission rate (vs. 8% placebo); 44% clinical remission rate	Supports high‐frequency multi‐donor FMT for active UC. [Suggestive]
	Rossen 2015, RCT	UC	Low‐frequency + single‐donor FMT	Subtraction	30.4% remission; no superiority vs. autologous	Low‐frequency single‐donor FMT has limited UC benefit [Negative]
	Cheng 2023, RCT	PD	Multi‐dose oral FMT capsules	Addition	MDS‐UPDRS score↓; improved cognitive/GI symptoms	Supports multi‐dose oral FMT for PD [Suggestive]
	Scheperjans 2024, RCT	PD	Single colonic FMT	Subtraction	No meaningful PD symptom improvement	Single‐dose FMT offers limited PD benefit [Negative]
	Wei 2015, case series	MRSA enterocolitis	Vancomycin pretreatment +3‐day FMT	Addition	100% remission; MRSA eliminated	Supports ‘pretreatment + multi‐dose FMT’ for MRSA enterocolitis [Suggestive]
	Singh 2018, proof‐of‐principle study	ESBL‐EB colonisation	Repeated FMT	Addition	Decolonisation rate↑with 2 FMTs vs. single FMT	Supports repeated FMT for ESBL‐EB decolonisation [Suggestive]
	Kang 2019, follow‐up study	ASD	FMT induction + maintenance	Addition	Sustained GI improvement; 47% ASD severity↓	Supports sequential FMT for long‐term ASD benefit [Suggestive]
	Holvoet 2021, RCT	IBS	Re‐transplantation for non‐responders/loss of response	Addition	67% response in initial responders	Supports retransplantation for IBS non‐responders [Suggestive]
	Bloom 2022, open‐label pilot trial	HE	High‐frequency oral FMT (5 doses/3w)	Addition	Dose‐dependent cognitive improvement	High‐frequency FMT needed for HE cognitive outcomes [Suggestive]
	Bajaj 2025, phase II RCT	HE	Low‐frequency FMT (1–3 doses/30d)	Subtraction	1‐dose recurrence 0%; > 1‐dose recurrence 17%	Supports low‐frequency FMT for HE recurrence prevention [Suggestive]
	Dutta 2014, prospective study	rCDI	Combined jejunal + colonic FMT	Addition	100% clinical resolution	Supports combined GI routes for rCDI cure [Suggestive]
	Bestfater 2021, retrospective study	rCDI	Bidirectional endoscopic FMT	Addition	100% D30/D90 cure and recurrence↓vs. single‐route	Supports bidirectional multi‐route FMT for rCDI [Suggestive]
	Ianiro 2022, meta‐analysis	Multiple indications (rCDI/IBD/MDRB, etc.)	Combined upper + lower GI FMT	Addition	Donor strain engraftment↑ vs. single route	Confirms multi‐route enhances colonisation across diseases [Suggestive]
	Lau 2024, prospective study	PACS with insomnia	Duodenal + colonic FMT	Addition	37.9% insomnia remission (vs 10% control)	Supports multi‐route FMT for PACS‐related sleep/anxiety [Suggestive]
	Paramsothy 2017, RCT	UC	Combined anatomical targeting + high‐frequency infusions	Addition	27% composite remission; comprehensive colonic coverage	Supports combined targeting for extensive UC lesions [Suggestive]
	Hvas 2019, RCT	UC	Distal colon‐targeted FMT via enema	Subtraction	24% clinical remission; effective for localised distal lesions	Supports distal enema for limited distal UC [Suggestive]
	Rossen 2015, RCT	UC	Upper GI‐targeted FMT via ND tube	Subtraction	No remission benefit; failed to cover extensive colonic lesions	Upper GI targeting insufficient for widespread UC. [Negative]
Post‐Mgmt	Wei 2016, RCT	UC	FMT + oral pectin	Addition	Mayo scores↓; delayed diversity loss	Supports pectin as post‐transplant lever for UC [Suggestive]
	Wu 2023, prospective study	T2DM	FMT + oral metformin	Addition	FBG/HbA1c↓; HOMA‐IR/BMI↓	Supports metformin as adjuvant for metabolic disorders [Suggestive]
	Allegretti 2024, RCT	IBD with rCDI	FMT + bezlotoxumab	Addition	No difference in recurrence/bacterial clearance	Bezlotoxumab addition offers no extra benefit. [Negative]
	Huttner 2019, RCT	MDRO colonisation	5‐day broad‐spectrum antibiotics + FMT	Addition	No decolonisation benefit; diarrhoea↑	Broad‐spectrum antibiotics lack efficacy. [Negative]
	Kedia 2022, RCT	UC	Multi‐donor FMT + anti‐inflammatory diet	Addition	60% clinical remission; 25% sustained deep remission	Supports anti‐inflammatory diet as post‐transplant lever for UC [Suggestive]
	Bryant 2021, case report	Refractory UC	FMT + low‐sulfur diet	Addition	Sustained remission; SCFA‐producing bacteria↑	Suggests low‐sulfur diet mitigates inflammation for refractory UC [Suggestive]
	Su 2022, open‐label trial	T2DM	FMT + PPW diet	Addition	FBG, HbA1c & BP↓; Bifidobacterium↑	Supports PPW diet as adjuvant for metabolic markers [Suggestive]
	Ng 2021, RCT	Obesity + T2DM	Repeated FMT + lifestyle intervention	Addition	Cholesterol/liver stiffness↓; probiotics↑	Supports lifestyle intervention to optimise FMT metabolic benefits [Suggestive]
	Ge 2016, pilot study	Slow transit constipation	FMT + high‐fermentable fibre	Addition	66.7% improvement; colonic transit time↓	Suggests high‐fermentable fibre enhances FMT for constipation [Suggestive]
	Mocanu 2021, phase 2 RCT	Metabolic syndrome	FMT + low/high‐fermentable fibre	Addition	Low‐fermentable: insulin sensitivity↑; high‐fermentable: no benefit	Supports low‐fermentable fibre for chronic metabolic disorders [Suggestive]

*Note:* Evidence type: [Suggestive]: These findings support hypothesis generation for further validation but are insufficient for definitive clinical recommendations; [Negative]: These findings guide avoidance of ineffective strategies in clinical practice and research design.

Abbreviations: Pre‐Donor (donor optimisation); Pre‐Recipient (recipient pretreatment); Pre‐Product (product engineering/preservation, pre‐administration); Peri‐Dose & Route (dose optimisation/combine route peri‐transplantation); Post‐Mgmt (recipient management post‐transplantation); GI (Gastrointestinal); AEs (Adverse Events); ARG (Antibiotic Resistance Gene); FBG (Fasting Blood Glucose); HbA1c (Glycated Haemoglobin); HOMA‐IR (Homeostatic Model Assessment for Insulin Resistance); BMI (Body Mass Index); MRSA (Methicillin‐Resistant *Staphylococcus aureus* ); ESBL‐EB (Extended‐Spectrum β‐Lactamase‐Producing Enterobacterales); MDRO (Multidrug‐Resistant Organism); PACS (Post‐acute COVID‐19 Syndrome); MDS‐UPDRS (Movement Disorder Society‐Sponsored Revision of the Unified Parkinson's Disease Rating Scale); wk (week); mo (month);↑ (increase); ↓ (decrease).

## 3 Improvements in Pre‐Transplantation

To enhance conventional FMT, pre‐transplant optimisation emphasises the modulation of the donor microbiome and preparation of the recipient gut environment. This strategic approach aims to facilitate the efficient colonisation and functional expression of transplanted microbiota.

### 3.1 Donor‐Side Optimisation

#### 3.1.1 Dietary Interventions: Constructing Functional Microbiota

The efficacy of FMT is critically dependent on the stability, functionality, and colonisation capacity of donor microbiota. Key influencing factors include microbial circadian rhythms, which regulate host metabolic and detoxification processes (Thaiss et al. 2016). Additionally, dietary components, such as fibre, significantly affect the function of the microbiota by shaping its structure, notably through the regulation of short‐chain fatty acid (SCFA)‐producing bacteria (David et al. 2014; Thaiss et al. 2016). Furthermore, exercises such as running can significantly change the abundance of certain health‐promoting microbiota, highlighting the multifaceted nature of microbiota modulation (Zhao et al. 2018). Therefore, donor management should adhere to the principles of rhythmic stability, structural integrity, and functional compatibility, which can be achieved through targeted ‘additive and subtractive strategies’.

##### 3.1.1.1 Addition Strategy

This approach aims to augment beneficial flora and their functions. For instance, increasing the intake of soluble fibre from whole grains and vegetables stimulates the growth of fibre‐degrading bacteria, such as *Faecalibacterium prausnitzii* and *Eubacterium rectale* , thereby enhancing the production of SCFAs. Leibovitzh et al. (2024) demonstrated that a 14‐day anti‐inflammatory diet enriched *Paraprevotella clara* and *Prevotella copri* , which can drive the structure of the recipient's flora to approximate the characteristics of the donor. This change may be related to the reduction in inflammatory indicators in the recipient after transplantation, thereby confirming the diet‐driven enhancement of anti‐inflammatory microbiota.

##### 3.1.1.2 Subtraction Strategy

The objective is to mitigate harmful bacteria and their associated metabolic pathways by limiting the prolonged consumption of high‐fat and high‐sugar foods. The previously referenced UCED study indicated that restricting sulfur‐rich pro‐inflammatory foods suppressed sulfur‐metabolising pathways (e.g., L‐methionine and L‐cysteine) and decreased the abundance of pro‐inflammatory bacteria, such as *Flavonifractor plautii* and *Streptococcus parasanguinis* . These shifts result in reduced production of inflammatory metabolites, such as hydrogen sulfide, thereby promoting anti‐inflammatory outcomes in FMT.

However, the research (Smits et al. 2018) found that transferring microbiota from vegetarian donors to recipients with metabolic syndrome presents challenges in achieving functional improvements. Although the recipients' microbiota shifted toward the characteristics of the vegetarian donor, key indicators such as trimethylamine N‐oxide levels and markers of vascular inflammation showed no significant changes. This suggests that diet‐induced shifts in microbial composition may not directly translate into functional benefits. Two factors may explain this outcome: insufficient duration/intensity of dietary intervention to eliminate harmful microbial components and recipients continuing an omnivorous diet post‐transplant, which counteracts donor microbiota regulation. Therefore, effective FMT necessitates the simultaneous dietary management of both the donor and recipient to ensure the functional stability of the transplanted microbiota. Conventional FMT studies often focus solely on the transplantation procedure while neglecting post‐transplantation management. In contrast, the proposed AMT hypothesis emphasises full‐cycle microbiota transplantation therapy, which is a major advantage of this paradigm.

Currently, no studies have investigated the optimisation of donor microbiota through exercise or pharmacological interventions. Future research should explore whether moderate exercise regimens or targeted pharmacological agents can modulate donor microbial diversity and functional potential, thereby enhancing FMT efficacy.

#### 3.1.2 Precise Regulation of Bacterial Liquid Component

Careful management of the bacterial population composition is essential for improving the safety and efficacy of FMT. This is primarily achieved through a strategy that incorporates both the addition of beneficial components and mitigation of potential risks.

##### 3.1.2.1 Addition Strategy

This strategy involves supplementing bacterial suspensions with nutritional additives, specific enzymes, vitamins, metabolites, or drugs to enrich beneficial metabolites or enhance targeted physiological functions. Notably, enriching/purifying specific functional microbial communities in donor suspensions is also a core ‘addition’ strategy. Studies have indicated that utilising donor faeces enriched with Bifidobacterium significantly improves the efficacy of FMT in patients with irritable bowel syndrome *(IBS)* (Mizuno et al. 2017). Additionally, a preclinical study by Chen et al. demonstrated that the inclusion of a composite protective agent (5% sucrose + 5% inulin + 1% cysteine hydrochloride) in donor bacterial suspensions preserved anaerobic viability and promoted recipient colonisation, thereby validating the targeted additive optimisation of bacterial liquid components (Chen et al. 2025). These findings underscore the importance of donor screening and in vitro enrichment of beneficial taxa in FMT. However, simply adding probiotics, such as Lactobacillus, into bacterial mixtures did not significantly enhance donor microbiota colonisation or clinical outcomes in patients with rCDI (Garza‐Gonzalez et al. 2019), highlighting the necessity for a comprehensive evaluation considering the donor's baseline microbiota characteristics, strain properties, and dosage.

##### 3.1.2.2 Subtraction Strategy

This strategy systematically removes potential risk factors from bacterial suspensions, including pathogens, toxins, and physical or chemical contaminants. WMT employs multi‐step centrifugation and washing techniques to effectively eliminate undigested residues, fungi and pro‐inflammatory metabolites. Evidence indicates that WMT reduces adverse event rates compared to conventional FMT in animal studies and shows efficacy in various diseases (Chen, Liu, et al. 2020; Chen, Tian, et al. 2020; Zhang et al. 2020, 2023; Liang et al. 2024; Xiao et al. 2025). Another classic subtractive approach is SER‐109, a purified spore‐based formulation derived from donor faeces: ethanol treatment eliminates non‐spore‐forming live bacteria, while retaining beneficial spore‐forming Firmicutes. This selective removal reduces the risk of pathogens and ensures product consistency, underscoring the value of targeted subtraction in microbial preparation (Feuerstadt et al. 2022; Straub et al. 2024). Furthermore, processing conditions are crucial, as oxygen exposure severely impairs the survival and function of obligate anaerobic bacteria. A randomised double‐blind study (Costello et al. 2019) demonstrated that anaerobically processed donor suspensions achieved a higher 8‐week steroid‐free remission rate (32%) than aerobically processed samples (9%) in patients with mild‐to‐moderate ulcerative colitis (UC), with some maintaining remission for up to 12 months, confirming the necessity of anaerobic conditions. Additionally, a ‘subtractive’ strategy, which involves lowering glycerol levels, should be utilised for the preservation of bacterial suspensions. Although glycerol is a common cryoprotectant, high concentrations inhibit anaerobic function and increase the risk of transplantation failure. A 10% glycerol concentration provides an optimal balance between maintaining bacterial viability and minimising inhibitory effects (Benech, et al. 2025). However, excessive subtractions require careful consideration. In rCDI, lyophilised FMT was more effective than sterile faecal filtrate without live bacteria (Kao et al. 2025). This evidence underscores that the therapeutic effect of FMT depends on microbial communities, and subtraction strategies should be restricted to eliminating clearly defined risks while preserving core microbes.

#### 3.1.3 Precision Strategies: Engineered Microbiota and Super Donors

To address the challenges of conventional FMT and meet the increasing demands for standardisation and regulatory compliance, two innovative strategies have emerged: synthetic microbiota transplantation (SMT) and super‐donor transplantation.

First, the super‐donor strategy emphasises the identification of individuals with highly complex, synergistic, and functionally complete natural microbiomes. A super donor is a healthy individual whose faecal microbiota induces substantial clinical improvement in most recipients. Its efficacy is derived from the ecological benefits of the overall microbiome structure rather than from individual strains. Research has demonstrated that utilising a single super donor significantly enhances symptom response and quality of life in patients with IBS (El‐Salhy et al. 2020). In contrast, mixed‐donor FMT yields inconsistent results. For instance, a clinical trial (Johnsen et al. 2018) reported reduced effectiveness, while another (Halkjaer et al. 2018) found no significant improvement in patients with IBS. This indicates that mixing donors does not produce an additive effect; rather, competitive interactions among communities from different donors may hinder the colonisation and synergy of critical microbial groups in the recipient gut. These findings underscore the ecological value of a well‐structured microbiome and the unique efficacy of super donors.

Another innovative strategy is SMT, which achieves precise intervention through the in vitro assembly of microbial communities with defined functions. Fundamental research has validated the feasibility of this strategy; for instance, Liu et al. constructed synthetic microbial consortia for CDI and demonstrated therapeutic efficacy in a murine model, highlighting the ‘subtraction’ of uncertain components and the ‘addition’ of functional strains within a defined community (Liu et al. 2023). Clinically, multiple studies (Kao et al. 2021; Rode et al. 2021; Menon et al. 2025) on rCDI have demonstrated that combined strain preparation produced through standardised industrial fermentation of faeces from healthy donors improves cure rates and reduces recurrence. These findings not only confirm the clinical efficacy of SMT but also underscore its technical strengths: consistent batch production, reduced donor reliance, and lower pathogen risk through industrial‐scale processes, thereby offering a scalable and precise therapeutic option. However, the advancement of SMT depends on a deeper functional understanding of key bacterial strains and accurate identification of effective microbial consortia for specific diseases. For instance, algorithms such as iMic can be employed to assess the recipient status and optimise strain combinations (Shtossel et al. 2023).

Building upon the concept of defined consortia in SMT, a further precision strategy involves the use of engineered live biotherapeutic products (eLBPs). This approach employs an the ‘addition‐subtraction’ logic at a genetic level. The core ‘addition’ entails equipping a selected bacterial host strain with novel, therapeutic capabilities, such as the sustained production of beneficial metabolites like butyrate (Bai and Mansell 2020), effectively programming it to function as an in situ producer of therapeutic metabolites within the gut. Concurrently, a ‘subtraction’ strategy is employed at the genetic level to optimise the bacterial host by knocking out competitive or undesirable metabolic pathways (e.g., those producing acetate or promoting autolysis) to enhance therapeutic efficiency and safety (Wang et al. 2022). Preclinical studies in models such as inflammatory bowel disease and metabolic syndrome have demonstrated that engineered strains can effectively modulate host physiology, including improvements in insulin sensitivity (Wang et al. 2022) and reinforcement of the intestinal barrier (Kang et al. 2024; Wu et al. 2024).

In summary, the super‐donor and SMT strategy represent two distinct approaches to achieving microbial ‘addition and subtraction’ in AMT. The super‐donor strategy capitalises on the intrinsic ecological synergy of intact native microbial communities, whereas SMT achieves standardisation through deliberate ‘subtraction’ by eliminating uncertain components and ‘addition’ by introducing specific functional bacteria. Building on SMT, engineered microbiota further extends this paradigm by employing genetic design to enhance therapeutic functions precisely while minimising the risks of pathogenicity or off‐target effects. These strategies are inherently complementary: the super‐donor approach leverages natural ecological advantages, whereas SMT and engineered microbiota emphasise controllability, standardisation, and precision. This evolution toward precision microbial engineering reflects a broader trend in synthetic biology. As reviewed by Jin et al., this field is accelerating the transition from defined microbial consortia to engineered bacteria consortia for targeted disease diagnosis and therapy. Collectively, such progress supports the development of high‐precision microbial editing tools and next‐generation, GMP‐oriented microbiota products for clinical applications (Jin et al. 2025).

### 3.2 Recipient‐Side Optimisation

Recipient pretreatment aims to enhance the efficacy of FMT by modulating the gut environment and reducing ecological resistance. This is accomplished through both additive strategies, such as synergistic drug combinations, and subtractive strategies, which involve the selective reduction of harmful or competitive microbes.

#### 3.2.1 Addition Strategy

Recipient pretreatment with drugs aims to enhance the efficacy of FMT through synergistic effects on the gut microbiota. However, current studies show considerable variability in outcomes, which are influenced by the disease context, medication type, FMT protocol, and individual patient characteristics. A study on patients with UC found that a 3‐week course of prednisolone prior to FMT did not significantly improve donor microbiota colonisation or clinical remission rates compared to placebo (van Lingen et al. 2024). Nonetheless, subgroup analyses from a meta‐analysis (Gefen et al. 2025) indicated that UC patients previously treated with biologics or methotrexate before FMT showed higher clinical and endoscopic remission rates than those without such treatments, suggesting that certain drugs may act synergistically with FMT.

Evaluating the synergistic effects of pretreatment poses several challenges. Many patients have already received baseline treatments, and discontinuing medications to establish a ‘pure FMT’ control group raises ethical issues. Currently, no universally effective pretreatment protocol exists for drug‐FMT combination therapy. Future studies should adopt more rigorous designs, such as accounting for baseline medication use and establishing appropriate controls, to clarify the mechanisms and conditions for specific drug‐FMT synergy, thereby advancing its clinical application.

#### 3.2.2 Subtraction Strategy

It entails the pretreatment of recipients by selectively reducing harmful or competitive gut microorganisms. This approach diminishes ecological resistance and enhances the colonisation and functionality of the donor microbiota, thereby improving the efficacy of FMT.

Keshteli et al. analysed nine studies with 118 UC patients, revealing that the antibiotic‐pretreated FMT group had a significantly higher clinical remission rate (54.0%) than the non‐pretreated group (25.1%, *p* = 0.03; Keshteli et al. 2017). A large‐scale study (Chen, Liu, et al. 2020; Chen, Tian, et al. 2020) involving 1501 patients with various indications further confirmed that both antibiotic‐only and antibiotic‐plus‐intestinal‐cleansing pretreatments improved clinical outcomes compared to no preparation (*p* = 0.001) without increasing adverse events. The ‘Consensus of Chinese Experts on Gut Microbiota and Fecal Microbiota Transplantation in Inflammatory Bowel Disease (2025 Edition)’ explicitly recommends antibiotic pretreatment before FMT for IBD patients with *Clostridium difficile* infection and/or small intestinal bacterial overgrowth (Parenteral, Enteral Nutrition Branch of the Chinese Medical, A., Chinese Society for the Promotion of Human Health, S., Technology, Committee on Gut, M., and Fecal Microbiota Transplantation, S.P.M.A 2025). This approach operates through several key mechanisms: elimination of pathogenic organisms to reduce interference with the donor microbiota, decrease in the density and diversity of resident commensals to reduce ecological competition and facilitate engraftment, and modulation of the intestinal microenvironment to promote a more favourable niche for the transplanted microbiota.

## 4 Improvements in Transplantation Process

### 4.1 Optimisation of Treatment Frequency and Mixed Donor Bacterial Suspensions

#### 4.1.1 Addition Strategy

Recent studies indicate that chronic conditions requiring sustained microbial management, such as IBD, Parkinson's disease (PD), IBS, and autism spectrum disorder (ASD), benefit from increasing the frequency and duration of FMT or adopting a sequential transplantation strategy (e.g., maintenance therapy after remission). This additive approach progressively introduces healthy microbiota, counteracts colonisation resistance, and promotes ecological stability in the gut. Clinical evidence supports its efficacy: in a study on UC (Paramsothy et al. 2017), high‐frequency FMT (40 sessions over 8 weeks) with multi‐donor material achieved higher remission rates than low‐frequency protocols (Rossen et al. 2015; Hvas et al. 2019), while maintenance therapy effectively reduced relapse rates (Haifer et al. 2022). Similarly, multi‐dose oral capsule FMT administered over 3 weeks improved motor and cognitive symptoms in individuals with PD (Cheng et al. 2023; Scheperjans et al. 2024). Extended or repeated FMT demonstrated greater effectiveness in decolonising antibiotic‐resistant bacteria (Wei et al. 2015; Singh et al. 2018). Poor initial responders with IBS may benefit from additional treatments or higher doses (Holvoet et al. 2021), and structured FMT in ASD has led to sustained behavioural improvement and microbial stability (Kang et al. 2019). Furthermore, a meta‐analysis indicated that multi‐donor mixed bacterial solutions are superior to single donors in inducing remission of UC (Levast et al. 2023).

In contrast to the high cure rate of single‐dose FMT for rCDI, chronic diseases require sustained reconstruction of a stable microbial ecosystem, highlighting the necessity of persistent treatment. Therefore, a phase‐specific strategy (tailoring frequency and intensity to disease progression and individual response) is essential for long‐term therapeutic success.

#### 4.1.2 Subtraction Strategy

In the AMT system, ‘subtraction’ reflects the deliberate reduction of transplant frequency. This approach systematically decreases the intervention intensity to avoid potential risks and burdens associated with redundant operations while ensuring core therapeutic benefits. The optimisation process focuses on three key aspects: reducing healthcare costs and financial burdens, alleviating patient discomfort and compliance pressures stemming from repeated invasive procedures, and preventing disruption of the recipient's gut microbiome due to frequent interventions. However, this strategy is not universally applicable and must be tailored to individual patient conditions, specific disease pathologies, and clearly defined treatment objectives. For example, in a study on treating hepatic encephalopathy (Bajaj et al. 2025), it was discovered that an increased frequency of FMT did not confer additional clinical benefits or enhance safety in recurrence prevention compared to a single transplant. Conversely, another study (Bloom et al. 2022) reported that multiple FMT sessions (3–5) could improve cognitive function in a dose‐dependent and sustained manner. This inconsistency underscores the necessity of aligning frequency reduction with specific treatment objectives. If frequent interventions are deemed critical for achieving primary endpoints, then arbitrary reductions may compromise efficacy.

In conclusion, transplant frequency optimisation requires balancing risks and efficacy with precise alignment of disease stage, severity, and treatment goals. Both arbitrary reductions and excessive transplant frequencies do not constitute optimal treatment approaches.

### 4.2 Combined Administration Routes

Combined multi‐route administration addresses the limitations of single routes, such as gastric acid degradation in the upper gastrointestinal tract and rapid colonic clearance in the lower tract. Consequently, it extends intestinal coverage and prolongs microbiota‐mucosa interaction time, thereby significantly improving the colonisation efficiency and spatial distribution of the donor microbiota.

Numerous clinical studies have validated this additive approach. In patients with post‐acute COVID‐19 syndrome and insomnia, improvements in sleep and anxiety symptoms were observed after FMT via simultaneous gastroscopy and colonoscopy (Lau et al. 2024). A meta‐analysis (Ianiro et al. 2022) further confirmed that combined delivery enhances the success of microbial engraftment. In cases of rCDI, pan‐gastrointestinal transplantation protocols achieved higher cure rates than single‐route interventions (Dutta et al. 2014; Bestfater et al. 2021), underscoring the therapeutic advantages of multi‐route administration.

Effective implementation requires a personalised design based on anatomical targeting. Research on UC offers critical insights: Paramsothy et al. achieved comprehensive colonic coverage and a 27% composite remission rate by utilising colonoscopy‐targeted proximal colon delivery in conjunction with enema‐based distal administration (Paramsothy et al. 2017). Similarly, Hvas et al. (2019) reported a 24% clinical remission rate using enemas that targeted only the distal colon. However, Rossen et al. (2015) found no significant benefit from nasoduodenal tube delivery, as it failed to cover extensive colonic lesions. These findings emphasise the importance of aligning administration routes with lesion distribution: widespread colonic involvement necessitates both proximal and distal delivery, whereas localised distal lesions may be adequately managed with enemas alone.

This additive strategy optimises the spatial complementarity and temporal continuity. Disease‐tailored sequential regimens can further improve clinical outcomes. For instance, in constipation‐predominant IBS, initial enema delivery may stimulate peristalsis and optimise the local microenvironment, followed by oral bacterial capsules to achieve phased intervention. This anatomically and pathologically informed integration offers a technical pathway to overcome traditional FMT colonisation bottlenecks, highlighting the value of AMT in refining therapeutic protocols for microbiota‐related diseases. However, the most appropriate path must also consider patient preferences, tolerance, disease characteristics, and physician experience.

## 5 Improvements in Post‐Transplantation

### 5.1 Drug Synergy With **FMT** : Strategies and Challenges

Combination drug strategies to enhance FMT efficacy are being explored; however, their synergistic outcomes remain variable and context‐dependent. Factors such as drug characteristics, underlying diseases, FMT methodologies, and individual patient differences influence the success of the treatment. Therefore, a critical evaluation of combination approaches must consider both supporting evidence and reported failures.

Certain combinations exhibit synergistic effects. Adjunctive metformin use with FMT in metabolic disorders achieves greater improvements in fasting blood glucose levels and HbA1c than FMT alone (Wu et al. 2022). In UC, the addition of supplemental pectin has been shown to reduce Mayo scores and enhance the efficacy of FMT (Wei et al. 2016), primarily by promoting microbial engraftment and sustaining microbial diversity post‐transplantation.

However, not all drug combinations yield the expected synergistic outcomes. A multicenter *randomised controlled* trial (Allegretti et al. 2024) demonstrated that the addition of bezlotoxumab to FMT in patients with IBD and rCDI did not enhance recurrence prevention, bacterial clearance, or symptom relief compared to FMT alone. This indicates that once microbiota restoration is achieved, combination drug therapy may offer no further advantage. This suggests that additive strategies should focus on profound synergistic interactions rather than simple combinations of agents. Moreover, certain combinations can be counterproductive; for example, broad‐spectrum antibiotics with FMT disrupt the commensal microbiota and impair microbial restoration, thereby diminishing colonisation efficacy and increasing adverse events (Huttner et al. 2019). This underscores that within an additive strategy, non‐selective destructive actions can negate or even reverse potential therapeutic benefits.

In conclusion, the combined effect of drugs and FMT (additive approach) is not guaranteed. Therefore, combination strategies must be rigorously justified when FMT alone demonstrates high efficacy. The evaluation of any drug used in combination with FMT must follow three principles: solid evidence‐based foundations, a clear mechanism of synergy, and benefits outweighing potential risks. Irrational drug combinations may *diminish* therapeutic outcomes and introduce unnecessary risks and costs, contradicting the principles of precision medicine.

### 5.2 Enhancing FMT With Diet and Lifestyle Interventions

Post‐FMT recipient management emphasises lifestyle and dietary adjustments to promote a stable microenvironment for transplanted microbiota. This approach uses targeted nutritional strategies to improve the physicochemical conditions of the gut and enhance clinical outcomes. Evidence indicates that such integrative approaches are effective in treating metabolic disorders (MD) and IBD.

In MD (Bloom et al. 2022), the combination of FMT with lifestyle interventions significantly improved lipid profiles and reduced liver stiffness in patients with obesity and type 2 diabetes mellitus (T2DM). These benefits are attributed to increased probiotic abundance, underscoring the importance of microenvironment modulation in enhancing microbial metabolic function. Furthermore, the probiotics‐prebiotics‐whole grains (PPW) diet (Su et al. 2022), when implemented alongside FMT, improves metabolic markers such as blood glucose and blood pressure. This synergistic effect results from the introduction of exogenous probiotics and the promotion of the expansion of beneficial native bacteria, including *Bifidobacterium* spp.

Dietary modifications are equally critical for IBD management. A low‐sulfur diet (Bryant et al. 2021) mitigates intestinal inflammation by inhibiting the production of hydrogen sulfide, thereby enhancing the efficacy of FMT in cases of refractory UC. Similarly, an anti‐inflammatory diet (Kedia et al. 2022), rich in polyphenols and omega‐3 fatty acids and low in saturated fats, improves immune homeostasis and increases the success rates of FMT in patients with mild‐to‐moderate UC.

Dietary fibre is an ideal synergistic substance; however, its efficacy may depend on the type of fibre selected. This selection necessitates precise screening based on the underlying disease context and the objective of maintaining colonisation stability. Low‐fermentable fibres enhance colonisation and insulin sensitivity by increasing stool bulk, modulating transit time, and improving microbiota‐mucus interactions (Ge et al. 2016; Mocanu et al. 2021). These characteristics render them suitable for the management of chronic conditions. Conversely, highly fermentable fibres rapidly produce SCFAs and inhibit pro‐inflammatory cytokines, such as tumour necrosis factor‐alpha and interleukin‐8 (Mocanu et al. 2021). However, their rapid fermentation can cause osmotic imbalances and gas production, which may compromise ecological stability with their long‐term use. Therefore, they are likely more appropriate for treating acute conditions.

In summary, the efficacy of post‐FMT dietary interventions relies on the strategic modulation of the microenvironment and personalised nutritional approaches. Customising these interventions to pathological characteristics, colonisation requirements, and risk–benefit balance represents a significant advancement toward individualised microbiota‐directed therapeutics in AMT.

## 6 From Mechanistic Plausibility to Translational Design

Preclinical studies provide biological plausibility for the core mechanistic rationale of the AMT framework and offer mechanistic rationale for its phase‐specific ‘addition–subtraction’ logic. For instance, studies have shown that microbiota from donors preconditioned through diet or exercise can transfer metabolic phenotypes to recipients, thereby supporting the concept of optimising the initial microbial ‘input’ (Lai et al. 2018). Further support comes from research combining donor preconditioning with probiotics supplementation and subsequent faecal microencapsulation has shown improved engraftment and functional outcomes, suggesting the synergy of multiple ‘addition’ strategies at the donor and product levels (Ba et al. 2025; Fan et al. 2026). On the manufacturing side, a critical ‘subtractive’ optimisation is exemplified by work establishing that anaerobic processing is essential for preserving the viability of obligate anaerobic bacteria, thereby improving product quality (Benard et al. 2023). In recipient studies, the synergistic interplay between preconditioning (e.g., antibiotic ‘subtraction’ to reduce ecological resistance) and transplantation protocol (e.g., multiple‐dose ‘addition’) has been highlighted in animal models as a key determinant of engraftment success (Gopalakrishnan et al. 2021). Finally, the full‐cycle logic of AMT is further supported by study where the benefits of a high‐fibre‐conditioned donor microbiota were significantly amplified when recipients consumed a compatible diet, effectively ‘feeding’ the transplanted microbes (Zhong et al. 2022).

Building on this preclinical foundation, we propose a hypothetical phase‐resolved AMT‐oriented trial‐design template for T2DM. This template may integrate coordinated ‘subtraction–addition’ operations across three stages: recipient preconditioning employs subtractive approaches to reduce harmful microbiota while preserving beneficial commensals. The donor/product optimisation focuses on community‐level functional enrichment rather than single‐strain replacement. Functional targets may draw on strain‐level evidence, such as butyrate‐producing *Faecalibacterium prausnitzii* (metabolic regulation via GLP‐1; Tolhurst et al. 2012) and *Akkermansia muciniphila* (improved insulin sensitivity via barrier reinforcement; Zhang, Kamm, et al. 2025; Zhang, Liu, et al. 2025). Peri‐transplant standardisation of delivery parameters coupled with post‐transplant dietary support to ensure engraftment and sustained function.

Notably, the diet‐modulated autologous FMT study conducted by Rinott et al. serves as a compelling clinical exemplar of this whole‐process logic, despite not being designed as an AMT trial (Rinott et al. 2021). The study employed a two‐phase, self‐donor design. In the initial weight‐loss phase, a green‐Mediterranean diet functionally ‘reprogrammed’ the participant's microbiota; stool collected at the weight‐loss nadir was then processed into encapsulated formulations and administered back to the same individuals during a weight‐regain phase. This intervention, coupled with standardised delivery and continued dietary alignment, successfully attenuated microbiome deterioration, reduced weight regains, and mitigated insulin rebound specifically in the green‐Mediterranean group. This finding supports the feasibility of implementing AMT‐like whole‐process optimisation in clinical settings.

## 7 Concluding Remarks and Future Perspectives

### 7.1 Paradigm Shift: How Does **AMT** Redefine Microbiota Transplantation?

This review thoroughly illustrates the fundamental significance and prospective applications of AMT, which employs an ‘addition and subtraction strategy’ to refine the FMT. By systematically integrating optimisation strategies across the pre‐transplantation, transplantation, and post‐transplantation phases, AMT represents a paradigm shift from conventional empirical FMT practices to a precision medicine framework grounded in ecological and mechanistic principles.

Theoretically, the ‘addition–subtraction’ strategy reframes the therapeutic mechanism of FMT not only as changes in microbial abundance but also as an active reconstruction of functional ecological niches. Its success depends on donor‐recipient compatibility, gut environmental adaptability, and precisely timed interventions, such as stepwise transplantation aligned with disease progression, which emphasises the co‐evolution between community functional capacity and host microenvironment stability. Clinically, this paradigm necessitates a shift in clinical practice from a standalone transplantation event to a personalised long‐term management process. By integrating optimised dietary and lifestyle support, a new FMT paradigm for chronic diseases, such as IBD and metabolic syndrome, is expected to be established.

Conventional FMT focuses excessively on the transplantation phase alone, neglecting the overall management of the treatment process, which fails to address niche resistance and ensure long‐term microbial stability. In contrast, AMT systematically integrates the pretreatment of both donors and recipients to optimise the quality of the donor bacterial solution and reduce the recipient ecological resistance. It employs a stepwise frequency and multipath combined transplantation approach to promote colonisation, alongside postoperative microenvironment regulation to maintain stability.

### 7.2 Standardisation Needs for AMT: Insights From Reporting Deficiencies

This review is the inaugural application of the PRIM guidelines to evaluate the quality of reporting in contemporary FMT clinical research. In Chapter 2, the bibliometric analysis and PRIM quality assessment demonstrated that FMT research is rapidly transitioning from case reports to clinical trials. The PRIM evaluation identified six major reporting deficiencies, highlighting the fundamental challenges in the standardisation of traditional FMT. This emphasises that future AMT studies must adhere strictly to established reporting guidelines, such as PRIM, to establish transparent and reproducible reporting structures, thereby enhancing both technical reproducibility and the reliability of research outcomes.

Moreover, the application of AMT necessitates the establishment of supportive, technical standards and policy frameworks. The production of microbial preparations requires strict standardisation of anaerobic operating conditions, cryoprotectant concentrations, and excipient compositions. Multi‐omics technologies should be used during donor screening to comprehensively evaluate the functional integrity and ecological resilience of microbial communities. Furthermore, regulatory systems must develop specialised evaluation pathways tailored to innovative products, such as SMT, complemented by dynamic surveillance platforms that track antimicrobial resistance gene transfer, pathogen reactivation, and metabolic perturbations, alongside standardised adverse event reporting aligned with PRIM guidelines. This regulatory evolution must address the core challenge of transitioning advanced therapies from a medical technology model to a pharmaceutical development paradigm. Importantly, AMT does not require the regulation of all FMT modalities as pharmaceuticals. Conventional donor‐derived FMT, which relies on non‐standardised, donor‐specific materials, is appropriately overseen as a medical procedure or technology. In contrast, defined or engineered microbial consortia—with well‐characterised components, standardised manufacturing, and predictable efficacy—should be classified under medicinal product frameworks (e.g., investigational drugs, live biotherapeutics). This distinction justifies stepwise, proportionate regulatory oversight enhancement, aligned with the growing complexity and standardisation of AMT therapies.

### 7.3 Future Directions: Key Challenges and Avenues for Breakthroughs in AMT

Current studies have several limitations. Many trials are characterised by small sample sizes and short follow‐up periods. Additionally, drug combination studies often inadequately control for baseline medication variables. Furthermore, analyses of gut microbiota predominantly rely on 16S rRNA sequencing, which limits insights into metabolic functions. There is also a notable regional bias, as the data primarily originate from Western populations, leaving dietary and geographic influences underexplored. The screening criteria for super‐donors lack racial diversity, which may restrict their global applicability. Current FMT research tends to overemphasise the role of bacteria while neglecting the contributions of phages and fungi. This review may also omit non‐English literature and recent studies. Much of the evidence has been derived from observational studies and small randomised controlled trials (RCTs).

Future efforts should prioritise four key areas: (1) the development of real‐time in situ metabolic imaging to monitor transplanted microbiota, combined with organoid–microbiota co‐cultures for high‐throughput screening of beneficial microbes and drugs; (2) the advancement of precision editing tools (e.g., phage/CRISPR‐Cas) for targeted gene insertion or removal, alongside the establishment of GMP‐grade SMT production focusing on strain survival and delivery stability; (3) the creation of comprehensive evaluations of super‐donors that integrate metagenomics, metabolomics, host immune markers and community‐level ecological/functional network analyses; (4) the construction of intelligent decision‐support systems that incorporate baseline microbiota, disease characteristics, and lifestyle data for dynamic protocol adjustments, including responsive transplantation frequency. Additionally, further applications should be explored in the context of neurodegenerative disorders and tumour immunotherapy and validated through large, multi‐regional cohort studies. At the same time, we recognise that practical implementation will also depend on health‐economic feasibility. The resource demands stem from core interconnected components: rigorous donor screening and management (including targeted preconditioning), standardised microbial preparation optimisation, recipient preconditioning, and peri−/post‐transplant monitoring with adjunctive therapies—all of which require substantial time, labor, and technological inputs. To balance resource constraints with therapeutic accessibility, a tiered implementation strategy is warranted: standardised baseline donor screening, microbial processing, and essential recipient safety assessments should serve as universal minima; while higher‐cost, higher‐complexity components (e.g., personalised donor preconditioning and advanced post‐transplant monitoring) should be reserved for high‐risk recipients, refractory cases, or investigational contexts where enhanced precision is justified.

## Author Contributions

Haojia Lin and Xiaocang Cao contributed to the conception and design of the review. Haojia Lin wrote the first draft of the manuscript. Jun Huang, Qiuyue Tu, and Zelin Feng conducted the literature search and screening. Huizhen Li, Yanru Zhang, Xinyue Wei, Qinghua Yi, and Hetong Zhang conducted data extraction. Haojia Lin, Yu Wang, Xiaoqin Li, and Anjiang Wang accessed report quality (follow the PRIM guidelines). Haojia Lin and Zehan Chen performed data analysis. Yueting Li and Hongtian Shentu created all the figures and tables. Xiaocang Cao, Ye Chen, and Xiaolong He critically reviewed the manuscript, provided advice, and approved the final manuscript. All the authors have read and approved the final manuscript.

## Funding

This review was supported by the National Key R&D Program of China (grant number 2024YFA1307102), Shenzhen Medical Research Fund (C2501011), Shenzhen Key Laboratory of Gastrointestinal Microbiota and Disease, Shenzhen Science and Technology Program (Strategic Emerging Industries Special Project) (ZDSYS20220606100800002), Shenzhen Science and Technology Program (Young Scientists Project in Industrial Fields)(LCYSSQ20220823091405012), and Sanming Project of Medicine in Shenzhen (SZSM202411029).

## Conflicts of Interest

The authors declare no conflicts of interest.

## Supporting information


**Figure S1:** Literature screening process.
**Figure S2:** Pie chart of PRIM score distribution.

**Table S1:** The missing items of the included studies based on the initial PRIM.

**Data S1:** Literature retrieval strategy.

## Data Availability

The authors confirm that the data supporting the findings of this study are available in the article and its Supporting Information.
