# Supplementary material for: Advance Microbiota Transplantation: A Novel Addition–Subtraction Paradigm for Optimising Faecal Microbiota Transplantation
Source: Microb Biotechnol. 2026 Mar 10;19(3):e70323. doi: 10.1111/1751-7915.70323 (PMC12972834; doi:10.1111/1751-7915.70323)
Supplement: Supplementary file 1 — Figure S1: Literature screening process. Figure S2: Pie chart of PRIM score distribution. [file MBT2-19-e70323-s001.docx]

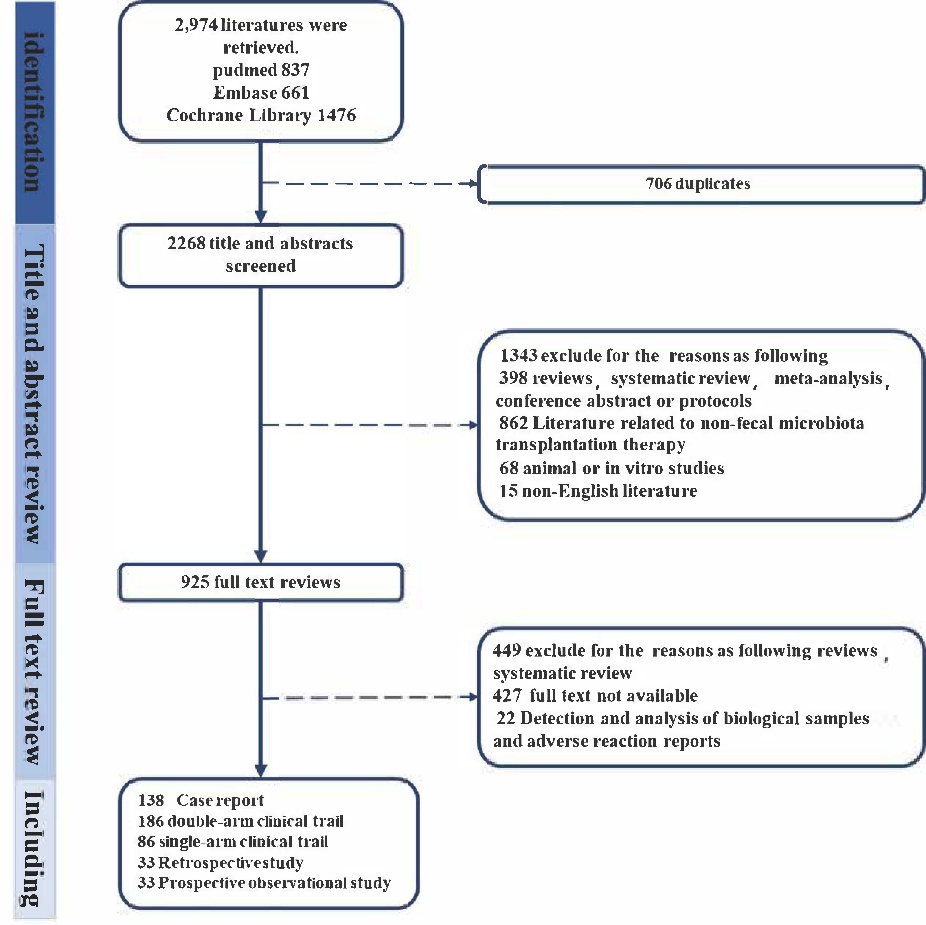


**Supplementary Figure 1. Literature screening process.**


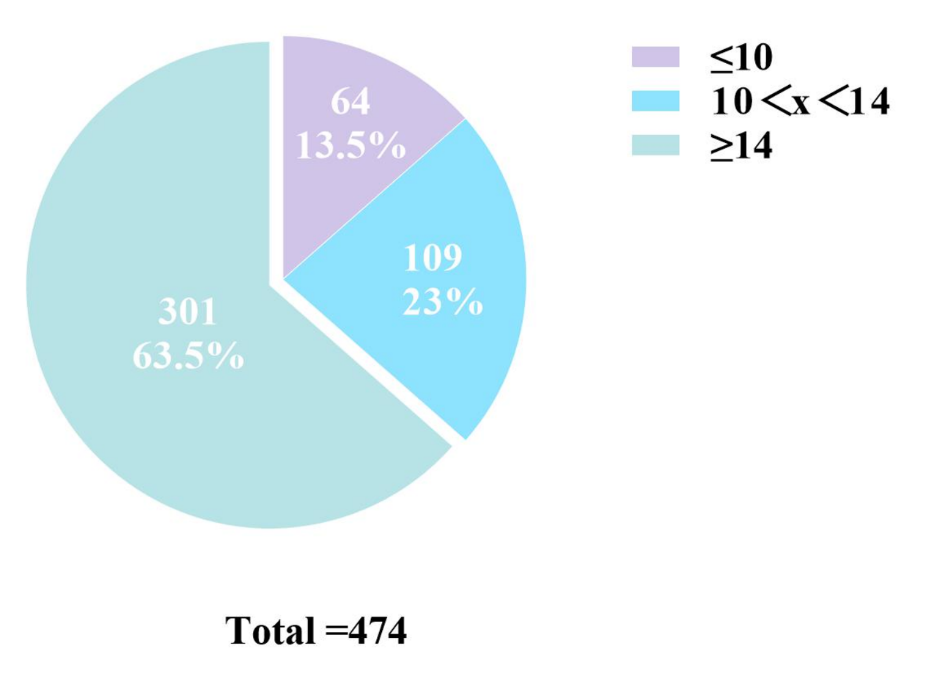


**Supplementary Figure 2. Pie chart of PRIM score distribution.**
