# Supplementary material for: Advance Microbiota Transplantation: A Novel Addition–Subtraction Paradigm for Optimising Faecal Microbiota Transplantation
Source: Microb Biotechnol. 2026 Mar 10;19(3):e70323. doi: 10.1111/1751-7915.70323 (PMC12972834; doi:10.1111/1751-7915.70323)
Supplement: Supplementary file 2 — Table S1: The missing items of the included studies based on the initial PRIM. [file MBT2-19-e70323-s002.docx]

| **Items** | **Description** | **Total**  **（N=409）** | **Case**  **（N=140）** | **Clinical trail**  **（N=269）** | **P value** |
| --- | --- | --- | --- | --- | --- |
| Indication | Diagnosis Disease condition | 0（0.0）  24（5.9） | 0（0.0）  2（1.4） | 0（0.0）  22（8.2） | —  **＜0.01** |
| Delivery route | Delivery route | 14（3.4） | 7（5.0） | 7（2.6） | 0.2528 |
| Source | Source | 20（4.9） | 12（8.6） | 8（3.0） | **＜0.05** |
| Preparation for products | Screening methods | 141（34.5） | 75（53.6） | 66（24.5） | **<0.0001** |
|  | Producing methods | 154（37.7） | 68（48.6） | 86（32.0） | **＜0.01** |
| Classification | Legal and ethical statement Classification for the products | 53（13.0）  264（64.5） | 31（22.1）  112（80.0） | 22（8.2）  152（56.5） | **<0.0001**  **<0.0001** |
| Dosage | Dose | 101（24.7） | 39（27.9） | 62（23.0） | 0.3337 |
|  | Frequency | 8（2.0） | 5（3.6） | 3（1.1） | 0.1290 |
| Formulation | State Subsidiary material | 115（28.1）  234（57.2） | 56（40.0）  89（63.6） | 59（21.9）  145（53.9） | **＜0.001**  0.0733 |
| Concomitant treatment | Patient preparation | 129（31.5） | 66（47.1） | 63（23.4） | **<0.0001** |
|  | Treatments affecting the outcome | 127（31.1） | 44（31.4） | 83（30.9） | 0.9108 |
| Efficacy | Definition  Rate or data available for the rate calculation | 51（12.5）  27（6.6） | 29（20.7）  19（13.6） | 22（8.2）  8（3.0） | **＜0.001**  **<0.0001** |
| Safety | Severity and management of adverse events | 87（21.3） | 43（30.7） | 44（16.4） | **＜0.01** |
|  | Rate or data available for the rate calculation | 94（23.0） | 43（30.7） | 51（19.0） | **＜0.01** |

Supplementary Table 1. The missing items of the included studies based on the initial PRIM.
