# Supplementary material for: Advance Microbiota Transplantation: A Novel Addition–Subtraction Paradigm for Optimising Faecal Microbiota Transplantation
Source: Microb Biotechnol. 2026 Mar 10;19(3):e70323. doi: 10.1111/1751-7915.70323 (PMC12972834; doi:10.1111/1751-7915.70323)
Supplement: Supplementary file 3 — Data S1: Literature retrieval strategy. [file MBT2-19-e70323-s003.docx]

**Literature retrieval strategy**

| Database/Platform | Search Query | Filters | Results |
| --- | --- | --- | --- |
| Pudmed | Fecal Microbiota Transplantations or Fecal Microbiota Transplant or Fecal Microbiota Transplants Fecal Microbiome Transplantation or Fecal Microbiome Transplantations or Fecal Transplant or Fecal Transplants or Donor Feces Infusion or Donor Feces Infusions Fecal Transplantation or Fecal Transplantations or Intestinal Microbiota Transfer or Intestinal Microbiota Transfers or Intestinal Microbiota Transplantation or Intestinal Microbiota Transplantations or Intestinal Microbiome Transplantation or Intestinal Microbiome Transplantations or Intestinal Microbiota Transplant or Intestinal Microbiota Transplants or Intestinal Microbiome Transfer or Intestinal Microbiome Transfers or Fecal Microbiota Transfer or Fecal Microbiota Transfers or Intestinal Microbiome Transplant or Intestinal Microbiome Transplants | in the last 10 years, Adaptive Clinical Trial, Case Reports, Clinical Study, Clinical Trial, Clinical Trial Protocol, Clinical Trial, Phase I, Clinical Trial, Phase II, Clinical Trial, Phase III, Clinical Trial, Phase IV, Comparative Study, Controlled Clinical Trial, Corrected and Republished Article, Multicenter Study, Observational Study, Pragmatic Clinical Trial, Randomized Controlled Trial | 837 |
| Embase | ('fecal microbiota transplantation'/exp OR ('bacteriotherapy (feces)'/exp OR 'bacteriotherapy (feces)' OR 'faecal bacteriotherapy'/exp OR 'faecal bacteriotherapy' OR 'faecal enema'/exp OR 'faecal enema' OR 'faecal infusion'/exp OR 'faecal infusion' OR 'faecal matter transplant'/exp OR 'faecal matter transplant' OR 'faecal microbial transplant'/exp OR 'faecal microbial transplant' OR 'faecal microbial transplantation'/exp OR 'faecal microbial transplantation' OR 'faecal microbiome transplant'/exp OR 'faecal microbiome transplant' OR 'faecal microbiome transplantation'/exp OR 'faecal microbiome transplantation' OR 'faecal microbiota transplant'/exp OR 'faecal microbiota transplant' OR 'faecal microbiota transplantation'/exp OR 'faecal microbiota transplantation' OR 'faecal transplant'/exp OR 'faecal transplant' OR 'faecal transplantation'/exp OR 'faecal transplantation' OR 'fecal bacterial transplant'/exp OR 'fecal bacterial transplant' OR 'fecal bacterial transplantation'/exp OR 'fecal bacterial transplantation' OR 'fecal bacteriotherapy'/exp OR 'fecal bacteriotherapy' OR 'fecal enema'/exp OR 'fecal enema' OR 'fecal infusion'/exp OR 'fecal infusion' OR 'fecal instillation'/exp OR 'fecal instillation' OR 'fecal matter transplant'/exp OR 'fecal matter transplant' OR 'fecal matter transplantation'/exp OR 'fecal matter transplantation' OR 'fecal microbe transplant'/exp OR 'fecal microbe transplant' OR 'fecal microbial transplant'/exp OR 'fecal microbial transplant' OR 'fecal microbial transplantation'/exp OR 'fecal microbial transplantation' OR 'fecal microbiome transplant'/exp OR 'fecal microbiome transplant' OR 'fecal microbiome transplantation'/exp OR 'fecal microbiome transplantation' OR 'fecal microbiota transplant'/exp OR 'fecal microbiota transplant' OR 'fecal microbiotal transplant'/exp OR 'fecal microbiotal transplant' OR 'fecal microflora transplantation'/exp OR 'fecal microflora transplantation' OR 'fecal transfusion'/exp OR 'fecal transfusion' OR 'fecal transplant'/exp OR 'fecal transplant' OR 'fecal transplantation'/exp OR 'fecal transplantation' OR 'feces bacteriotherapy'/exp OR 'feces bacteriotherapy' OR 'feces microbe transplantation'/exp OR 'feces microbe transplantation' OR 'feces microbiota transplantation'/exp OR 'feces microbiota transplantation' OR 'feces microflora transplant'/exp OR 'feces microflora transplant' OR 'feces microflora transplantation'/exp OR 'feces microflora transplantation' OR 'fmt (fecal microbiota transplantation)'/exp OR 'fmt (fecal microbiota transplantation)' OR 'gut microbial transplant'/exp OR 'gut microbial transplant' OR 'gut microbial transplantation'/exp OR 'gut microbial transplantation' OR 'gut microbiome transplant'/exp OR 'gut microbiome transplant' OR 'gut microbiome transplantation'/exp OR 'gut microbiome transplantation' OR 'gut microbiota transplant'/exp OR 'gut microbiota transplant' OR 'gut microbiota transplantation'/exp OR 'gut microbiota transplantation' OR 'gut microflora transplantation'/exp OR 'gut microflora transplantation' OR 'imt (intestinal microbiota transplantation)'/exp OR 'imt (intestinal microbiota transplantation)' OR 'intestinal microbe transplantation'/exp OR 'intestinal microbe transplantation' OR 'intestinal microbiota transplant'/exp OR 'intestinal microbiota transplant' OR 'intestinal microbiota transplantation'/exp OR 'intestinal microbiota transplantation' OR 'intestinal microflora transplantation'/exp OR 'intestinal microflora transplantation' OR 'rectal bacteriotherapy'/exp OR 'rectal bacteriotherapy' OR 'stool enema'/exp OR 'stool enema' OR 'stool infusion'/exp OR 'stool infusion' OR 'stool instillation'/exp OR 'stool instillation' OR 'stool microbial transplantation'/exp OR 'stool microbial transplantation' OR 'stool transplant'/exp OR 'stool transplant' OR 'stool transplantation'/exp OR 'stool transplantation' OR 'fecal microbiota transplantation'/exp OR 'fecal microbiota transplantation')) AND [2016-2025]/py AND 'clinical trial'/it |  | 661 |
| Cochrane Library | (Fecal Microbiota Transplantation toFecal Microbiota Transplantations or Fecal Microbiota Transplant or Fecal Microbiota Transplants Fecal Microbiome Transplantation or Fecal Microbiome Transplantations or Fecal Transplant or Fecal Transplants or Donor Feces Infusion or Donor Feces Infusions Fecal Transplantation or Fecal Transplantations or Intestinal Microbiota Transfer or Intestinal Microbiota Transfers or Intestinal Microbiota Transplantation or Intestinal Microbiota Transplantations or Intestinal Microbiome Transplantation or Intestinal Microbiome Transplantations or Intestinal Microbiota Transplant or Intestinal Microbiota Transplants or Intestinal Microbiome Transfer or Intestinal Microbiome Transfers or Fecal Microbiota Transfer or Fecal Microbiota Transfers or Intestinal Microbiome Transplant or Intestinal Microbiome Transplants):ti,ab,kw with Cochrane Library publication date Between Jan 2016 and Jul 2025, in Trials |  | 1476 |
|  |  |  |  |
|  |  |  |  |
